# Exergaming: A Good Exercise Option During the Coronavirus Pandemic

**DOI:** 10.7759/cureus.43799

**Published:** 2023-08-20

**Authors:** Michael Paalani, Margaret Bredehoft, Susanne Montgomery, Ruben Chipuli, Hildemar Dos Santos

**Affiliations:** 1 Public Health, Loma Linda University School of Public Health, Loma Linda, USA; 2 Public Health, Public Health Services, Santa Ana, USA; 3 Public Health, Loma Linda University, Loma Linda, USA; 4 Preventive Care, Loma Linda University Medical Center, Loma Linda, USA; 5 Preventive Care, Loma Linda University School of Public Health, Loma Linda, USA

**Keywords:** physical activity, rate of perceived exertion, exercise metabolic rate, metabolic equivalents, exergaming

## Abstract

The purpose of this study was to evaluate Wii sports programs as a potential physical activity solution to increasing inactivity among young adults and potentially improving the immune system. An evaluation was done on five Wii Sports games, including tennis, baseball, bowling, golf, and boxing. Measures included caloric expenditure and metabolic equivalents (MET) expended. A total of 16 subjects participated in two interactive video game sessions that took place over a 14-day period, in which their caloric expenditure (based on metabolic equivalents and rate of perceived exertion [RPE]) was compared with accepted reference values associated with playing the actual sports. Results showed that Wii sports were associated with higher MET values than published norms for other actual sports, and all Wii sports showed caloric expenditure that was four to ten times higher than traditional sedentary gaming. In conclusion, the results justify the integration of interactive video-gaming technologies into future fitness programs targeted toward young adults and, consequently, decrease the risk of metabolic syndrome and obesity.

## Introduction

Increasing rates of obesity have become a significant public health problem in the US and worldwide and often result from modern conveniences and technological advances that continue to facilitate more sedentary lifestyles [1-3]. However, it may be possible to utilize one of the gaming industry's latest advances in interactive gaming, or exergaming, to our advantage. Several studies have shown that playing active video games significantly increases energy levels beyond those seen in sedentary states [4-7]. In addition, it is important to look at relative perceived exertion and delve into the social cognitive components associated with exergaming, which can enhance motivation and enjoyment for physical activity and ultimately provide psychological and cognitive benefits, including positive changes in exercise self-efficacy and self-esteem [8-10]. Therefore, active video games may be effective as a therapeutic tool for promoting optimal health and reducing obesity risk for adults by providing benefits such as reductions in cholesterol, hemoglobin A1c, and body fat [6].

Exergaming may be an alternative solution to meet the daily physical activity recommendation for specific populations, as observed among girls in Saudi Arabia [11]. Furthermore, although many studies have demonstrated both physical and cognitive benefits of exergaming among children and adolescents, there is limited research on the effects of active video games on the adult population. Finally, it is possible that exergaming may contribute to improved health and immunity, based on research showing the benefits of exercise and moderate physical activity on cardiorespiratory system fitness and enhanced immune function among an elderly population [12]. Other studies have demonstrated that taking just a brisk walk can reduce the number of sick days by 50% over a 12- to 15-week period (when compared to engaging in no exercise) [13]. Therefore, these studies support the idea that exergaming can promote immune system efficiency when compared to sedentary behavior.

The objective of this study was to measure the caloric expenditure (and metabolic equivalents [METs]) of individuals participating in interactive video games and compare those values with accepted reference values associated with playing actual sports. Reference values were obtained from the update to the Compendium of Physical Activities [14].

## Materials and methods

Study population

Participants had to be 18 years of age or older to be included in the study, and those who had a medical/health condition that would serve as a contraindication for engaging in physical activity were excluded. Participants were recruited in person and given an informed consent letter to complete. Participants also received the opportunity to ask questions and/or decline participation if they were not interested. This study was reviewed and approved by the California State University San Bernardino Institutional Review Board (IRB # 09032).

Sampling technique

This experimental study utilized a convenience sample of 16 healthy adults aged 21-45, consisting of nine males and seven females who had limited exposure to the Wii Sports game. Each subject participated in two sessions that took place over a 14-day period. The group's average age was 34.25 ± 5.01, and the average BMI for the group was 25.97 ± 4.20.

Data collection

During session one, participants received a formal explanation of the purpose of the study. Consent forms were finalized and signed prior to data collection. Baseline demographics included resting heart rate, height and weight measurements, baseline exercise metabolic rate (EMR), and rate of perceived exertion (RPE). All subjects consented to participate and were required to pass a submaximal graded exercise test to ensure adequate physical fitness. They were also given time to become familiar with the metabolic cart apparatus and develop proficiency by working through the training modules of each Wii sports game.

Baseline EMR was assessed while participants were on a treadmill performing the submaximal graded exercise test using the Bruce protocol. Participants were connected to a Polar heart rate monitor strapped around their chest, along with a mask connected to a new A calibrated, mobile metabolic cart (New Leaf City), which measured exhaled air. Following this assessment, each participant made an appointment to return for session 2 of the study within the next 14 days.

During session 2, participants were asked to engage in 10 minutes of gaming on each of the five Wii sports (tennis, boxing, baseball, golf, and bowling). The order in which the games were played was randomly assigned. Prior to the onset of testing, resting heart rates were taken to establish a baseline. Wearing the same mask used during the EMR test, exhaled air (O_2_ and CO_2_) was collected and analyzed via the metabolic cart to determine energy utilization during the game sessions. At the end of each 10-minute gaming session, participants were given water for hydration and allowed 10 minutes to recover before continuing to the next sport.

Outcome measures

The outcome measures for the study were energy expenditure in calories, METs, and RPE. To assess the degree of caloric burning and metabolic equivalents expended, descriptive statistics using the mean ± standard deviation were used to summarize the RPE and MET values for each of the five Wii Sports. To assess if exergaming activities produced energy expenditure comparable to published norms, mean scores were used to compare the energy expenditure of the observed data with those published in the Updated Compendium of Physical Activities for the actual sports (tennis, baseball, bowling, golf, and boxing).

Power analysis

A power analysis was performed using a one-sample t-test with a two-sided significance level of 0.05, a power of 0.8, and a null mean of 3. It was determined that 15 subjects were needed to ensure sufficient power, and since 16 subjects were included in the study, sufficient power was met.

## Results

For the five Wii sports, 10 minutes of boxing had the highest MET value (4.56 ± 1.37), whereas golf had the lowest MET value (1.82 ± 0.51). Table 1 presents the aggregate results as well as individual results according to gender based on caloric expenditure (cal/kg/hr) and METs for each of the five Wii sports.

**Table 1:** Comparison of metabolic equivalents and caloric expenditure for five Wii Sports

	All participants	Males	Females
Metabolic equivalent and caloric expenditure by Wii Sports category			
Baseball	2.96 ± 0.89	3.32 ± 0.96	2.50 ± 0.55
Bowling	2.47 ± 0.68	2.70 ± 0.66	2.18 ± 0.63
Boxing	4.56 ± 1.37	4.89 ± 1.23	4.13 ± 1.52
Golf	1.82 ± 0.51	2.03 ± 0.32	1.54 ± 0.60
Tennis	3.32 ± 0.83	3.34 ± 1.10	3.30 ± 0.30
Average caloric expenditure (Cal/kg/hr) for each Wii Sport category			
Baseball	2.39 ± 0.58	2.46 ± 0.60	2.30 ± 0.57
Bowling	1.86 ± 0.45	1.71 ± 0.38	2.04 ± 0.50
Boxing	4.24 ± 1.45	4.31 ± 1.31	4.14 ± 1.73
Golf	1.54 ± 0.55	1.46 ± 0.52	1.64 ± 0.60
Tennis	3.13 ± 0.94	2.66 ± 0.77	3.74 ± 0.81

Individuals were also asked to report their RPE for the five sports following each 10-minute session. Again, boxing was associated with the highest RPE (6.03 ± 1.66), and golf was associated with the lowest RPE (2.40 ± 0.89). Table 2 summarizes the RPE values while playing each of the five Wii sports. In general, the RPE scores were consistent with light to moderately heavy exertion. Moreover, RPE assists individuals in determining their self-reported level of effort, and can help improve practice adherence [15].

**Table 2:** Reported rate of perceived exertion following five Wii Sports *Using BORG's CR10 scale

	All participants	Male	Female
Average rate of perceived exertion*			
Tennis	4.64 ± 1.69	4.52 ± 1.56	4.79 ± 1.97
Boxing	6.03 ± 1.66	6.11 ± 0.83	5.93 ± 2.44
Baseball	3.94 ± 1.48	3.74 ± 1.27	4.19 ± 1.79
Golf	2.40 ± 0.89	2.22 ± 0.73	2.62 ± 1.08
Bowling	3.43 ± 1.28	3.41 ± 1.11	3.45 ± 1.57

**Figure 1:**
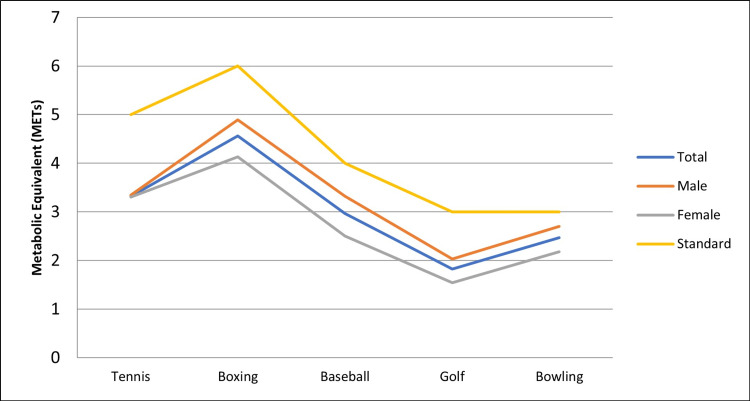
Comparison of metabolic equivalents observed while playing Wii Sports compared with published norms

Lastly, the calculated MET values during exergaming were compared to published norms for playing the actual sport, utilizing the 2000 update to the Compendium of Physical Activities. The results showed that males playing baseball (3.3 METS versus the reference value of 4.0 METS) and males playing bowling (2.7 METS versus the reference value of 3.0 METS) were the only two sports that were comparable to published norms for the actual sport. All other results were statistically significantly lower than published norms. Table 3 summarizes the mean difference and 95% confidence intervals of METs observed for each of the Wii games compared with the published norm. Figure 1 shows the trend for the published norm (standard) compared with the results for all the study participants (total) and for each gender-specific subgroup (males and females).

**Table 3:** Mean difference and 95% confidence interval of metabolic equivalents of each game type compared to the standard (reference value) ^1^The reference values taken from the compendium were: tennis (5.0, tennis, doubles play), boxing (6.0, boxing, punching bag), softball (4.0, softball officiating), golf (3.0, golf, miniature, driving range), and bowling (3.0, bowling). *The result is statistically significant (1 sample t-test with p-values < 0.05) lower than the published norm. METs: metabolic equivalents.

	Ref. value^1^	All participants' METs absolute values/METs differences (CI)		Male METs absolute values/METs differences (CI)		Female METs absolute values/METs differences (CI)	
Tennis	5.0	3.32	−1.68 (−2.11, −1.24)*	3.34	−1.66 (−2.50, −0.81)*	3.30	−1.67 (−1.98, −1.42)*
Boxing	6.0	4.56	−1.44 (−2.17, −0.72)*	4.89	−1.11 (−2.06, −.017)*	4.13	−1.87 (−3.28, −0.46)*
Baseball	4.0	2.96	−1.04 (−1.52, −0.57)*	3.32	−0.68 (−1.42, 0.06)	2.50	−1.50 (−2.01, −0.99)*
Golf	3.0	1.82	−1.18 (−1.45, −0.91)*	2.03	−0.97 (−1.21, −0.72)*	1.54	−1.46 (−2.01, −0.91)*
Bowling	3.0	2.47	−0.53 (−0.89, −0.17)*	2.70	−0.29 (−0.81, 0.21)	2.18	−0.82 (−1.41, −0.24)*

## Discussion

Overall, this study's results are consistent with what is already known about interactive video gaming and enhanced energy expenditure over sedentary gaming. However, by comparing these results with the Compendium, it was apparent that the participant activity levels aligned with the norms of the actual sport reference values.

On average, brisk walking is associated with a MET value of 3.0 [14], whereas sedentary gaming has been shown to have a MET value of about 1.4 ± 0.06 [16]. From this study, Wii sports were not only comparable to brisk walking, but in some cases, they were associated with higher MET values than published norms for other actual sports (e.g., Wii boxing was nearly equivalent to actual tennis and had higher MET values than actual golf, bowling, and baseball). In addition, all Wii sports showed caloric expenditure that was four to ten times higher than traditional sedentary gaming.

All participants reported exertion levels ranging from light to moderately heavy intensity. RPE plays a strong role in one's attitude toward exercise. The Borg and CR10 scales have shown unwavering quality and legitimacy in healthy, clinical, and athletic adult populations [15]. One observation from the study was that the higher the RPE, the more engaged participants were in gaming. These results are promising and warrant additional research.

Exercise and physical activity may have an impact on the body's immune system. Home-based programs that use apps or devices such as smart watches can help track health-related data and improve quality of life. This will help healthcare providers assess patients' strengths and weaknesses in improving their overall health status [12]. Research indicates that the preventive effect of exercise can be credited to its anti-inflammatory impact (when done regularly), which is associated with reduced visceral fat and heightened anti-inflammatory cytokines such as interleukin (IL)-1 receptor agonists and IL-10 [17]. Moreover, epidemiological and experimental research implies that moderate exercise will stimulate the replacement of immune cells between lymphoid tissues and circulation, which will help to improve immunosurveillance, host protection, and immune defense activity [13,18]. Lastly, reports have demonstrated that exercise training diminishes blood neutrophils in people with inflammatory conditions, indicating the possibility that exercise can function as an immune system regulator [17].

Limitations

Potential limitations of this study included previous exposure to Wii sports, engaging at varying levels of intensity, improper breathing or air leakage from the metabolic cart, and compendium data assumptions. However, a protocol was developed to screen those with limited Wii sports experience in order to observe and instruct participants to be engaged in the game. During session 1, participants were also given time to get acclimated to the oxygen mask. As for the compendium data, which includes published and accepted norms, limitations could not be addressed.

## Conclusions

The findings from this study and other similar studies underscore the need for more research in this area. If Wii Sports and other types of exergaming provide not only a good workout but also enhancement of mastery skills and increased self-confidence, it may be beneficial to motivate individuals to increase their physical activity level through exergaming. If exergaming is shown to be effective in encouraging other types of non-gaming physical activity, then it will be incumbent upon health educators to increase access to exergaming. In addition, based on evidence that individuals in clinical settings may benefit from exergaming as a way to improve general and disease-specific health outcomes, this alternative method of physical activity can be effective as a form of rehabilitation for those who are either homebound or unable to leave a hospital location. Additional populations are likely to benefit from exergaming as well, particularly those who are sedentary and show no interest in traditional exercise. In conclusion, future research should be done to determine whether exergaming can be tailored to fit the needs of the individual and whether their fitness level can be assessed to determine the optimal level of activity.
